# Strengthening mental health systems in Zambia

**DOI:** 10.1186/s13033-020-00360-z

**Published:** 2020-04-16

**Authors:** Margarate Nzala Munakampe

**Affiliations:** 1 Department of Health Policy and Management, School of Public Health, University of Zambia, Lusaka, Zambia; 2 Strategic Centre for Health Systems Metrics & Evaluations (SCHEME), Department of Epidemiology & Biostatistics, School of Public Health, University of Zambia, Lusaka, Zambia

**Keywords:** Mental health, Mental health systems, Mental health services, Barriers to service use

## Abstract

**Background:**

Studies in mental health care for low resource settings indicate that providing services at primary care level would significantly improve provision and utilisation of mental health services. Challenges related to inadequate funding were noted as significant barriers to service provision, with the contribution of low knowledge of mental health conditions and stigma in the community. This study aimed to explore the barriers to the use of mental health services in Zambia, suggesting health systems thinking approaches to solving these challenges.

**Methods:**

Primary data were collected through individual interviews from 12 participants; primary caregivers, health workers from public health institutions that treat mental health conditions and policymakers and implementers. The digitally recorded responses were transcribed and analysed using thematic analysis.

**Results:**

Key barriers to care included inadequate funding, few human resources, poor infrastructure and stigma. Barriers to care at policy, facility and individual or community level could be alleviated by strengthening the mental health system. Engagement of community health workers and increasing efforts to sensitise the community about mental health would prove beneficial.

**Conclusions:**

Strengthening the community health systems for mental health could improve access and increase utilisation of services.

## Background

Studies have suggested that providing services at primary care level would significantly improve the provision of community mental health services [1]. However, challenges related to inadequate funding are noted as significant barriers to service provision at that level, with the contribution of insufficient knowledge of mental health and mental health conditions in the community, stigma and the lack of prioritisation of cost-effective options from the policy level [2, 3]. Despite a global prevalence of 13%, inclusive of neurological and substance use disorders, mental disorders do not attract much attention [4]. The magnitude of mental disorders is amplified when comorbid with other disorders such as HIV/AIDS and coronary heart disease [5, 6]. Projections for this disease burden were set to increase to about 15 per cent by 2020 and depression producing the second most significant disease burden across all age groups.

In Zambia, the prevalence of mental disorders is approximately 20 per cent. Common mental disorders include acute psychotic episodes, schizophrenia, affective disorders, alcohol-related problems and organic brain syndromes [7]. The potential causes of mental health conditions include stressful family relationships, infections such as malaria, meningitis, syphilis and HIV, use, and the use and dependence on alcohol and other psychotropic substances [8–12]. Poverty increases susceptibility to mental health conditions [1]. About 60 per cent of Zambians are classified as poor [13]—poverty defined as the lack of access to employment, income, freedom on which goods and services to consume, and a lack of other basic needs [14]. A significant portion of individuals with severe and persistent mental health conditions live in rural areas [15].

Historically, mental health care has been a neglected part of the health system in Zambia, with services concentrated at provincial government hospitals and not at the primary care level [1]. There is low funding for activities at less than 1% of the national health budget [2]. A profile of mental health in Zambia [7], highlighting the arrangement of mental health care in the country, is described as ‘critical’. Key factors such as few trained human resources and the reliance on traditional medicine at the community level contribute to challenges of inadequate care. Services were also governed by the 1951 Mental Disorders Act, which was repealed. The Mental Health Act of 2018 is in place, but has not been operationalized yet.

Within the community, the stigma surrounding mental disorders is a significant barrier to mental health service utilisation [16]. In Zambia, students, detainees, prisoners, unemployed and others are described as ‘at risk of developing mental disorders’. However, only 15 per cent of them can use mental health services, and these are mostly students and prisoners, as they have targeted interventions and services [17]. Even among the covered, a majority of them face significant challenges as revealed in a study at an urban health centre in Lusaka, which showed that over 80 per cent of people face problems of poor accessibility and underutilisation [16].

Strengthening mental health systems in Zambia responds to the call to advance global mental health [18] by improving funding and enhancing monitoring of the mental health system in countries, taking services as close to people as possible. In light of this background, the main aim of this study was to find out the barriers to the utilisation of mental health services at three levels: policy, facility and individual level. A ‘systems thinking framework’ [19] is suggested, to understand these barriers and provide an indication of how to strengthen the overall mental health system in Zambia. While studies have looked into barriers at facility and individual level, such as stigma, seeking traditional healers and inadequate funding of the mental health activities [20–22], this study aims to add more information to the policy level dimension, as well as provide a response to the dearth of context specific information on mental health in Zambia.

## Methods

This paper reports findings from a qualitative case study, which was part of a mixed-methods study that aimed to investigate barriers to the utilisation of mental health services at the policy, facility, and community level. The case study collected data from the health facilities, while a cross-sectional survey collected data from the community.

The case study was conducted in 3 provinces; Lusaka, Ndola, and Kabwe, between August and December 2016. Five institutions, four public health institutions that treat mental health conditions and the Ministry of Health, were purposively selected for this study. Primary data were collected through individual interviews. An interview guide with open-ended questions was used to steer the discussions with the participants, administered by the principal researcher and one research assistant. Both took field notes throughout data collection. Interviews were only conducted after the researcher explained the study aims and procedures involved in the study.

A total of 12 participants were included in the study. A variety of information sources was introduced when selecting participants for the study; policymakers, health workers and the family members of the patients at the mental health facilities. Table 1 describes the study participants in some detail.

**Table 1:** Characteristics of the study participant (n = 12)

Participant	Location	Gender	Type
MH001	Kabwe	Female	Family member (main care-giver)
MH002	Kabwe	Female	Family member (main care-giver)
MH003	Kabwe	Male	Nurse
MH004	Lusaka	Female	Family member (main care-giver)
MH005	Lusaka	Male	Policymaker
MH006	Ndola	Male	Clinical Officer
MH007	Ndola	Female	Nurse
MH008	Ndola	Male	Family member (main care-giver)
MH009	Lusaka	Female	Family member (main care-giver)
MH0010	Lusaka	Male	Doctor
MH0011	Lusaka	Male	Policy maker
MH0012	Lusaka	Female	Nurse

All interviews were conducted in English, Bemba or Nyanja, and each interview lasted between 20 minutes and an hour, depending on the availability of the study participants. The digitally-recorded responses were transcribed and translated verbatim and read together with the field notes. Translated data were checked by the research assistant to ensure meanings were not lost. The data were managed and analysed using NVivo 10. Thematic analysis [23] was used as it was appropriate for, according to Braun and Clarke, 2006 [24] “analysing and reporting patterns (themes)” within data collected from the participants. The key themes were developed from a predetermined code structure, developed from the literature reviewed, highlighting barriers at policy, facility, and individual level in other studies. New themes emerged from the data at the three different levels, particular to the Zambian mental health care system. The coded data were triangulated with the field notes and other less formal discussions and impressions that were noted as the data were being collected.

Ethical clearance was obtained from the University of Zambia Biomedical Research Ethics Committee, UNZABREC (REF No. 013-06-15), and permission to carry out the study in the public health institutions was sought from the Ministry of Health, and the senior administrators at the health facilities. Individual written informed consent was obtained from all the participants before the interviews were conducted in private.

### Patient and public involvement

No patients who were clinically diagnosed with mental health conditions such as depression and substance abuse-related disorders at the health facilities were interviewed in this study. This study was conducted in three provinces in three districts; where the researcher worked closely with representatives from the district and provincial health offices, as well as the institutional administrative officers. The family caregivers of the patients were helpful and played an essential role in the study while caring for their family members.

## Results

The findings are presented under three main barrier domains: policy-level barriers, facility-level barriers, and individual-level barriers.

### Policy level barriers

#### Absence of an updated legal framework

Information collected from respondents at the policy level revealed barriers to effective implementation of the mental health policy. It was mentioned that reference the old Mental Disorders Act and implementation of the mental health policy was problematic because the two were not aligned on how to manage the patients. While the policy stipulated mental health management and care, the law reinforced the use of violence to manage the patients, usually with the help of the police. The bill had been under revision for over 10 years and was only passed in 2019. At the time of data collection, there was hope that the new law would allow for the management of patients to be responsive to current mental health policy rather than based on 1949 experiences and thinking.

*“The reading of the bill in parliament to enact the new laws that is the will of the government. So we expect that before, by the coming quarter next year* [2016]*, we might have a new law in mental health that will bring in better innovation in mental health.”* MH0011, Policymaker (Lusaka).

#### Budget constraints and inadequate allocations

Financial constraints also emerged as a barrier to adequate mental health services provision and the overall implementation of the mental health policy in Zambia. Budget limitations affected the provision of services in the community. One nurse from Kabwe had this to say:

*“We have in the past trained some Mental Health Assistants, but these have been based here at the facility. However, if the same people in the community were able to be trained and then share the knowledge with the rest of the community, I think that would be very useful. If there were an increase in the budget for mental health, then there would be an improvement”.* MH0001.

Also, the insufficient budget affected the training of mental health service providers. It was noted that the mental health sector attracts very little funding on its own, however linking mental health to other more prioritised diseases and conditions; and that attract more funding would be beneficial to mental health care.

*“*(…) *definitely the funding isn’t sufficient. It goes with what the WHO says, that it’s below 1%, like many other African countries. The budgetary allocation is not enough, and it usually has a ceiling. Last year it was about K300, 000. What can you do with that for national mental health service? We have a program to do with mental health and HIV. We are now trying to convince people in child health, maternal health to see if we can do a program on child and maternal mental health, it would help improve the health status of maternal mental health. The solution is riding on other services”.* MH0005, Policy Maker (Lusaka).

#### Drugs

At the primary care level, primary care kits with essential medicines needed to provide services were regularly provided to the health facilities. However, these kits did not contain drugs specific to mental health care. The lack of drugs was seen as a contributing factor to the many referrals at the provincial facilities from lower-level institutions. The Mental Health Policy stressed ‘bringing the services as close to the people as possible’, but this was not possible because it had not yet been effected at the lower levels of service provision. The health system did not provide services at the primary level, and the drugs were not provided. Besides, it was also noted that these drugs were very costly, and this affected access to drugs when the patients needed them, sometimes even at the higher-level institutions.

*“(*…) *some of the drugs are not available, and so we have to tell the relatives to go and buy for them. There are about five types of Antipsychotics, but ‘anti*-*side*-*effect’ drugs are not available. Even though we give the patients the prescriptions, the medicines are quite expensive”.* MH0007, Nurse (Ndola).

#### Management

The management of mental health services was noted as a barrier to the provision of mental health services in Zambia. Despite feeling that the mental health sector was neglected and not prioritised in terms of overall funding and the provision of medicines among other issues, health care providers felt that airing their views would not lead to any significant changes in their work. Thus, they continued working despite the challenging conditions.

*“I can blame this on management because to do these; we need money and transport* (…) *I feel there are no key people who can make decisions and implement them. If you have no voice, being heard is difficult, you continue working the way you work. Your voice has no impact, so you do things within your circles of influence”* MH0003, Nurse (Kabwe).

In terms of managing patients, the existing legal framework was seen as demeaning and discriminatory towards the patients, with terms such as ‘imbecile’ referring to the patients. It was also common practice for the members of the community to report any mental health-related case to the police first, usually because of the violence associated with some conditions. As a result, the lack of legal protection or support allowed many patients of mental conditions to be dragged to the mental health institutions against their will with the help of police officers, through the use of court orders. Some patients were antagonistic towards receiving any medication attached to such treatment. Hospitalisation in the old and dilapidated facilities also affected the re-socialisation of individuals after treatment as the community continued to remember them in that way even when they had received treatment and were better. A caregiver had this to say;

*“It is government policy for the police to come in because of the way he was he was scaring people so they cannot lock him up* (…) *until he stabilises and until the doctor says now he is okay. However, even with a court order, the police should treat him well, because he isn’t a criminal, it is an illness. You just talk to the person nicely”* MH000 4, Family Member (Lusaka).

While the facility and community level actors saw a failure in managing mental health services, on the policymaker’s side, however, it was reported that much had been done for mental health services; considering the changes that had happened over time, even though they also felt that there was more work to be done.

### Facility level barriers

#### Few experts or human resources

Generally, a shortage of human resources to meet the burden of mental health conditions in the country was reported by almost all the participants interviewed. The shortage was attributed to several reasons. Most of the health workers who decided to take the mental health career path ended up disinterested because they did not have many opportunities to develop their careers. When compared to HIV/AIDS management or maternal and child health, mental health providers did not have as many training opportunities as part of their continuous professional development. Some health workers were deployed at these facilities with basic mental health management knowledge but lacked opportunities to practice or see patients. They eventually diverting into other areas instead, and this was typical of many nurses and clinical officers. One nurse had this to say;

*“There is no motivation to work despite having knowledge and skill. There are no drugs, so we cannot treat the patient. There are no support staff like psychologists or social workers to come in using the multi*-*disciplinary approach. There should be all these people so that a patient is treated holistically. Demotivation is too much, despite having the knowledge* (…) *within PHC there is also the secondary level where someone is already sick, there should be a lot of manpower, social worker, psychiatrist a psychologist so that a patient can be treated holistically instead of just providing medication and allowing then to go”.* MH0003, (Kabwe).

#### Poor referral system

Ideally, the structure was that the clinics or health centres were supported by the zonal health centres (community health centres), and both were supported by the district hospitals which were in turn also supported by the provincial hospitals. Where the cases are complicated, the patients were referred to the district or provincial hospitals or the national specialist hospitals. However, this was not the case with the mental health referral system, as clients went directly to the provincial hospitals; at the mental health annexes or to Chainama Hills Hospital (the national mental health hospital) when the cases were more complicated or alarming for the caregivers. This was a severe breach of the system and thus led to overcrowding in the provincial hospitals and at the national specialist hospital. A nurse from Ndola (MH0007) said *“We receive very few referrals. People come straight to the hospital. Once they get detention orders, they come here and “dump” their patients here”.*

Despite the weak or “different” referral system, the family caregivers acknowledged that the health care providers were well trained as they were able to attend to them when they sought assistance. However, they were not sufficient to handle all their demands due to staff shortages. Training of Mental Health Assistants (Community Health Workers) helped alleviate the problem of insufficient human resources at Lusaka and Ndola hospitals. Conversely, the continuity was problematic due to insufficient budgetary allocation to mental health. A need for more personnel to be trained at the different levels of health care to support mental health programs was reported as a way to reach the community as these activities were already specified in the mental health policy.

### Individual-level barriers

#### Stigma

Stigma was one of the most significant barriers to the utilisation of mental health services noted at the individual or community level. Stigma was captured at three levels: self-stigma, stigma from family members and the community in which the patient lived, and stigma from the health care providers. Self-stigma came about when the patient was aware that they were sick, and they began to lose hope in medication and are from the family. A nurse from Ndola (MH0006) said *“(*…) *also self*-*stigma from the clients themselves it becomes very difficult for someone to recover because of certain beliefs they come with from the community”.*

The community was also reported to stigmatise patients with mental health conditions and this caused challenges related to adherence to medication and the general well-being of the patient. Even when the family took care of the patient, they could not protect them from the ridicule and judgment from their neighbours and friends. Finding out and being aware of the community’s stigma was reported to contribute to self-stigma as the patient was made aware of their condition.

*“There is a stigma in the community. Because they see you here at ward 12* [mental health ward] *and they even know that my husband is mad. Even when we are discharged when we reach the community, they are very mean; they call him “lishilu”* [a mad/foolish person] *“her husband is a mad man”. We may also try to hide from him, to hide the fact that he was here like when he asks me what brought him here I say its high blood pressure because of fear that the truth could cause a relapse. So people in the community are the ones that tell him what was wrong, that he had a mental health condition. So he sometimes feels too embarrassed to even come back here for medication”* MH0002, Family Member (Kabwe).

Sometimes clients or families were stigmatised because mental health conditions were seen as a sign of some involvement in the “supernatural” that had backfired. This form of judgment was founded in myths and misconceptions about the causes and implications of mental health conditions. Some misconceptions noted were that mental health conditions could be spread to other people through bites from patients and that mental health conditions were sexually transmitted. Community members were seen to propagate relapse as they remind the patient that they had a mental health condition.

While the health workers mentioned that most of the male patients were hospitalised due to substance abuse-related conditions, most female patients in the hospitals suffered from depression.

#### Education or awareness (Knowledge about mental health conditions)

Knowledge about mental health conditions was reported as vital in mental health care. All the family members acknowledged that before caring for a patient within their household, they had very little or no knowledge at all about mental health conditions. Having to face the condition through their family members made them more aware and knowledgeable. They also mentioned that knowing more about the conditions before their family members were afflicted would have improved their experience of caring for their family members. When a family caregiver was asked if she knew about the mental health condition before dealing it in her family, she had this to say;

*“No. I only knew after she* [her daughter] *was sick. I just used to look at people who are sick. I did not have any thoughts regarding mental health problems. I just worried about feeding my family and seeing to it that their needs were met. Because I am single. I did not know anything about these diseases”.* MH0001, Family Member (Kabwe).

## Discussion

The barriers to mental health service utilisation were investigated at the three levels; policy, facility and individual level. While inadequate financing for mental health affected most of the other health system domains, a ‘systems thinking’ approach [19], emphasises examining the different parts of the health system and how they all need to be prioritised to improve mental health in Zambia. The barriers are therefore discussed in terms of; leadership and governance of mental health and mental health services, mental health financing, human resources for mental health, mental health service provision, mental health commodities and information systems.

### Leadership and governance of mental health services

At the policy level, management was noted as one of the critical factors affecting the provision and utilisation of mental health services. Though management alone could not be blamed, it was cited as a key contributing factor, especially at policy and facility levels. Poor management was a barrier with regards to the prioritising of funds and actual budgetary allocation to the mental health sector. Due to the old and outdated Mental Disorders Act, which was still being reinforced [25] at the time of the study, the treatment that patients faced was that of criminals because that was stated in the law and the justice system enforced the law. It is hoped that the Mental Health Act of 2019 law will change the face of mental health in the country [26].

### Mental health financing

Regarding financing for mental health, the study found that the monetary allocation to the sector was too little to do much meaningful work. Priority was given to other diseases and disorders, and less than 1% of the health budget was allocated to mental health. These findings were consistent with studies in Ghana, Uganda and Sudan [20, 27]. Budgetary allocation directly affected the provision of drugs as shortages were reported in some instances, though some drugs were available. It also affected the management of services, as they could not be provided at the primary care level, particularly in the community. The plans (policies) were in place, but the finances were inadequate to implement them. The lack of sufficient funding also affected the physical state of the facilities. Most of the buildings were old or dilapidated and patients were stigmatized also based on the state these facilities. Similar findings were reported in other countries [18].

### Human resources for mental health

Inadequate funding, lack of prioritisation at the policy level, few human resources (specialists) led to a shortage of mental health services at primary care level. Because many health care providers opted to refer cases to the secondary or even tertiary facility, regardless of the severity of the case [28], this was noted as a form of stigma. Some of the cases seen at the secondary institutions could have been handled at the clinics and health posts, but because of lack of interest or experience in providing such services, the referrals took place. The inadequate funding also led to fewer opportunities for most of the health care providers to further their careers in mental health. The sector is not as well funded as other sectors such as HIV and adolescent health. As such, the health care providers did not choose mental health in their career progression. These findings were consistent with the studies done in Malawi related to mental health [28].

The lack of human resources [25] for mental health prevented the continued provision of Community Mental Health. Without such an intervention, many people are not aware of the importance of mental health, mental health conditions, and how to manage them.

### Service provision and commodities for mental health

A pattern of utilisation where services began at the secondary level, instead of primary care level emerged in the Zambian mental health system. Clients avoided the clinics as their first point of contact with the health system and went directly to the provincial hospitals when they sought care for the mental health conditions. Even when a health care provider was willing to provide services, the primary health care package had no medication to respond to the need for these services. These findings were not in line with those found in South Africa [29], as efforts were made to decentralise services and include them in the primary health care package.

### The impact of stigma

A health system cannot function without people. Therefore, the people who utilise these services must also be aware of and be able to access these services. However, individual-level barriers continued to affect access and utilisation. The main barrier at the individuals and community level was the stigma attached to mental disorders. Stigma was reported to be fueled by lack of knowledge or awareness about mental disorders as well as the state of facilities where these disorders were treated. Most studies also allude to this [30, 31]. This study highlighted stigma at three levels. The first was at the facilities where they accessed services, the second in the community and the third was ‘self-stigma’. Self-stigma was reported to be stigma the patient has towards himself and also due to adverse treatment from the people around.

Within the community, stigma towards the patients affected the pathway of treatment and in some cases may have contributed to relapse, as the patients found comfort in their old behaviour (particularly for substance-induced disorders). Other studies indicate that post-hospitalisation stigma is related to negative health outcomes for patients, including relapse [32] but can be combated by anti-stigma initiatives in the community [33]. The lack of provision of community mental health services from the health sector left little room for awareness and knowledge of mental health and mental health conditions in the community.

The study revealed that people who utilised services were more likely to be knowledgeable about mental health conditions are and that this was because most of the family members and patients only knew of these conditions after they encountered mental health conditions in the family. However, stigma still affected the management of these conditions in the community, as well as through the health system. Ultimately, financing of mental health care was seen as the solution to reducing stigma and increasing awareness of mental health conditions in the communities.

### Strengths and limitations

This study did not capture data on the functioning of health information systems as they relate to mental health. However, factors relating to how data is captured and used are crucial and could provide more information on the magnitude and burden of mental health conditions, the barriers related to specific mental disorders and impact overall management of the entire health system.

This study was carried out in selected locations; hence, transferability may be limited as these locations may have been representative of specific social contexts in the country. Since this information was acquired in government institutions, the private institutions may have had some insights that may not have been captured. Besides, the study excluded perspectives from patients themselves, but from their family caregivers as institutional permission to do so was not granted in time. The institutions included in the study required mental health practitioners from their institutions to interview the patients, instead of the research team. This was a requirement for conducting the research with patients, but could not be done within the study timeline. Nevertheless, the primary caregivers were capable of providing information on barriers to the utilization of mental health services in Zambia. This study focused on barriers at the policy, facility and individual level, but mostly on the supply side. Hence barriers in the community were not adequately captured.

Despite these challenges and limitations, the study collected data from 5 institutions in three provinces and these were different enough to transfer [34] information to most parts of the country with the same socio-economic and cultural landscape and created proxies for the situation in the other provinces, providing variation [34, 35] in study location needed to triangulate the findings. While theoretical saturation was not achieved in the study due to sample size limitations, variation and triangulation of data sources; the addition of the health workers, policy makers and implementers’ perspectives provided credible information that adds to the dearth of knowledge on mental health care in Zambia and the data generated is relevant for policy considerations. The findings are also a basis for more research on mental health in Zambia.

Also, the study adopted ‘well established and clearly exposed’ qualitative research methods and these added to the dependability or consistency of the methods [34–36]. While the sample was small and theoretical saturation was not achieved, the data was ‘relevant to society’ [35] as highlighted by Mays and Pope, providing credible information on barriers to utilisation of mental health services in Zambia. Overall, additional rigorous and scientifically valid studies on mental health care in Zambia are needed to fill-up the immense information gap in this sector.

## Conclusion

The study aimed to explore the barriers to the utilisation of mental health services in Zambia, and the mental health system is deeply affected by these barriers. A need to integrate mental health services into primary health care is one way to increase the accessibility and utilisation of mental health services. Some cases that were transferred to the provincial centres could have handled at the primary care level. Hence, integration at this level would improve the efficiency of the mental health system. Sensitising of health care providers about the need for mental health professionals, particularly at the community level was seen as a solution to increasing the human resource base for mental health, as well as providing incentives for career progression in the field. For the community members, awareness and community education efforts are needed to tackle the myths and misconceptions and stigma that perpetuates poor mental health management at the community level. Overall, strengthening the community health systems would improve access and increase utilisation of mental health services.

## Supplementary information

**Additional file 1.** Data collection tools: interview guides for the health workers, family care givers and policymakers.

## Data Availability

The data used or analysed during the current study are available from the corresponding author on reasonable request. The articles reviewed are available online.
