# Supplementary material for: Strengthening mental health systems in Zambia
Source: Int J Ment Health Syst. 2020 Apr 16;14:28. doi: 10.1186/s13033-020-00360-z (PMC7161303; doi:10.1186/s13033-020-00360-z)
Supplement: Supplementary file 1 — Additional file 1. Data collection tools: interview guides for the health workers, family care givers and policymakers. [file 13033_2020_360_MOESM1_ESM.docx]

**Data Collection Tools**

**Interview Guide (Nurses)**

**Barriers to the Utilisation of Mental Health Services in Zambia**

Interview Guide: Nurses

Date: ________________________

Interviewee Study ID: _______________________

Interviewer name: __________________________

Location: _________________________________

Age: Gender:

What do you think about mental health services in Lusaka/Ndola/Kabwe/Zambia?

What are the major challenges and obstacles in your opinion facing mental health care?

1. At the policy level

2. At the facility level.

3. At the individual level.

What factors do you think are contributing to the existing pattern of utilisation of mental health care?

What is the most common mental illness you diagnose?

How can we improve utilisation of mental health services? What areas do you want to see improved in mental health?

What are the challenges facing service provision in your facility?

From where do you receive mental health patients?

What do you think of integrating mental health services into primary care?

What do you think of training lay persons to treat people with mental illness?

What hinders your progress in the provision of care? Traditional healers, church?

**Interview Guide (Family members)**

**Barriers to the Utilisation of Mental Health Services in Zambia**

Interview Guide: Family Members

Date: ________________________

Interviewee Study ID: _______________________

Interviewer name: __________________________

Facility: _________________________________

Age: Gender: Occupation:

What do you think about mental health services in Lusaka/Ndola/Kabwe/Zambia?

What are the major challenges and obstacles in your opinion facing mental health care?

1. At the policy level

2. At the facility level.

3. At the individual level.

What factors do you think are contributing to the existing pattern of utilisation of mental health care?

Have you considered using alternative treatment? Traditional healers, church.

How has mental illness affected you financially as well as emotionally?

Do you feel there is sufficiently trained man power to handle service provision?

Do you feel stigmatised due to mental illness in your family? Health workers, community…

What did you know of mental illness before it started in your family?

What would you have done differently if you had known this information?

In your opinion, how can awareness about mental illness be increased?

**Interview Guide (Policy Makers)**

**Barriers to the Utilisation of Mental Health Services in Zambia**

Interview Guide: Policy Makers

Date: ________________________

Interviewee Study ID: _______________________

Interviewer name: __________________________

Facility: _________________________________

Age: Gender: Occupation:

What do you think about mental health services in Lusaka/Ndola/Kabwe/ Zambia?

What are the major challenges and obstacles in your opinion facing mental health services?

1. At the policy level

2. At facility level

3. At individual level.

What factors do you think are contributing to the existing pattern of utilisation of mental health care?

How might we improve the utilisation of mental health care?

How can you improve the linkage between mental health services and the health system?

What policies do you think are working and what are not in mental health?

What challenges have arisen in implementing these policies?

Do you think that it is possible to integrate mental health services into primary care?

Do you think that the Ministry of Health will be open to this approach?

How can more funds be raised for mental health care?

What do you think of mental health insurance?
